# Changes in healthy Wistar rat gut microbiome by short-term dietary cava lees intervention

**DOI:** 10.3389/fnut.2025.1641612

**Published:** 2025-09-17

**Authors:** Mercedes Berlanga, Alba Martín-García, Ricardo Guerrero, Montserrat Riu-Aumatell, Elvira López-Tamames

**Affiliations:** ^1^Departament de Biologia, Sanitat i Mediambient, Secció de Microbiologia, Facultat de Farmàcia i Ciències de l’Alimentació, Universitat de Barcelona, Barcelona, Spain; ^2^Departament de Nutrició, Ciències de l’Alimentació i Gastronomia, Grup d’Aroma i Factors de Qualitat Alimentària, Facultat de Farmàcia i Ciències de l’Alimentació, Campus de l’Alimentació de Torribera, Universitat de Barcelona, Santa Coloma de Gramenet, Spain; ^3^Departament de Patologia i Terapèutica Experimental, Laboratori de Microbiologia Molecular i Antimicrobians, Facultat de Medicina, Universitat de Barcelona, L'Hospitalet, Spain

**Keywords:** beta-glucans cava lees, healthy Wistar rat, metagenomics analysis, gut microbiota diversity, gut functionality

## Abstract

**Introduction:**

The gut microbiome plays a crucial role in host health through complex host–microbe interactions. Beta-glucans, structural polysaccharides found in yeast cell walls, have emerged as promising modulators of immune function and microbial ecology. Cava lees, a by-product of sparkling wine production composed of *Saccharomyces cerevisiae* cell walls, represent a rich source of beta-glucans that could be upcycled for nutritional and therapeutic applications.

**Methods:**

Twenty-four Wistar rats (12 males, 12 females) were randomly divided into control and treatment groups. The treatment group received daily doses of 2,000 mg lees/kg body weight for 14 days. Shotgun metagenomic analysis was performed to assess microbial composition and functional changes.

**Results:**

A 14-day cava lees supplementation study revealed significant shifts in gut microbiota composition and function. Baseline microbiota was dominated by Bacillota (64–72%) and Bacteroidota (23–32%) with sex-specific differences at the family level. Post-supplementation analysis showed increased Shannon diversity across both sexes, with beneficial enrichment of *Bifidobacteriaceae* and *Rikenellaceae* families and reduction of *Eubacteriaceae*. While global metabolic profiles remained stable, targeted functional pathways were significantly changed, including butyrate production genes. Females exhibited particularly elevated secondary bile acid modification genes (Mann–Whitney-Wilcoxon test *p* = 0.032), and male oxidative stress response pathways (Mann–Whitney-Wilcoxon test *p* = 0.016) showing both a potentially sex-dependent responses to dietary intervention.

**Conclusion:**

Working with healthy individuals provides a clear understanding of the normal, baseline microbiota composition and function before any intervention. These findings suggest a degree of plasticity of the gut microbiome and its responsiveness to dietary modifications. Beta-glucans from cava lees appear to create a favorable environment for beneficial bacteria, with sex-specific changes of certain bacterial families and functions. These findings provide a foundation for future translational research in humans. Nonetheless, to establish their true impact on human health, these observations in rodent models must be validated through appropriately designed human clinical studies.

## Introduction

The gut microbiome plays a fundamental role in host health, influencing metabolism, immune function, and even behavior through complex host–microbe interactions. Recent research has highlighted the potential of dietary interventions to modulate the gut microbiota composition and function, particularly through the administration of bioactive compounds. Among these, beta-glucans, which are structural polysaccharides found in the cell walls of yeasts and other fungi have emerged as promising modulators of both immune function and microbial ecology, shaping the gut microbial ecosystem by functioning as prebiotics (1).

Wine production, particularly in the elaboration of sparkling wines such as cava, generates significant quantities of cava lees as a by-product. These lees, composed of *Saccharomyces cerevisiae* cell walls, represent a rich source of beta-glucans that could potentially be upcycled for nutritional and therapeutic applications. These cell walls consist of beta-1,3-glucan (50–55%), beta-1,6-glucan (5–10%), mannoproteins (35–40%), and small amounts of chitin (1–2%) (2). Recently it was recognized the potential of cava lees as a valuable resource (3) Beta-glucans were approved as novel food ingredients by the European Commission in 2011, highlighting their potential in food applications, particularly in fermented products (4). Incorporating these fibers in foods can accelerate the fermentation process by lactic acid bacteria, resulting in a more pronounced reduction in pH, enhance food safety, and maintaining or even improving the sensory qualities of foods (5, 6).

The potential of cava lees as a source of dietary fiber has attracted considerable attention, particularly within the growing field of fiber–microbiota interactions. Most dietary intervention studies investigating this relationship employ controlled feeding trials, where participants consume specific types and amounts of dietary fiber over a set period. These studies commonly use techniques such as 16S rRNA gene sequencing or metagenomic analyses to assess changes in gut microbiota composition and function. To fully understand the impact of diet on the gut microbiota, it is essential to establish a baseline assessment of the microbiota in a healthy state, free from external influences such as previous dietary interventions. The gut microbiota is a highly complex ecosystem, comprising thousands of microbial species that vary significantly between individuals. “Healthy microbiota” refers to the stable and permanent members of the microbial community in healthy populations (7–9). A healthy gut ecosystem is characterized by a balance of cooperation and competition among its microbial members, which contributes to overall stability (10). Individual responses to dietary treatment can vary widely, influenced by both the composition and the metabolic capacity of each person’s resident gut microbiota, particularly regarding the ability to metabolize specific fiber supplements. As a result, dietary interventions often produce considerable inter-individual variability in physiological and microbiome responses, highlighting the complex interplay between diet, host factors, and the gut microbiome ecosystem (11–14).

Understanding the role of dietary fiber in shaping the gut microbiota may provide valuable insights for managing chronic diseases linked to the gut microbiome (11). Human studies undoubtedly yield the most directly relevant data, but preclinical animal models—particularly rodents—offer critical experimental advantages for mechanistic and longitudinal investigation. Mice and rats are frequently used due to their genetic tractability, ability to exert a control over environmental, dietary, and pharmacological variables, thereby minimizing confounding factors that are difficult to standardize in human cohorts (12). Despite these strengths, it is important to acknowledge key anatomical and physiological differences among mice, rats, and humans. For instance, rodents have a substantially higher metabolic rate, smaller body size, and distinct feeding behaviors, including nocturnal grazing and coprophagy, all of which can impact gut microbial ecology (12, 15). At the compositional level, the predominant bacterial phyla—Bacillota and Bacteroidota—are shared across humans and murine models, and many genera overlap. However, significant quantitative differences exist at the genus and species level (15). Even within the same rodent species, environmental factors such as housing, diet, and provider often exert a pronounced effect on their gut microbiota composition than genetic background (12). As in mice, the rat gut microbiota evolves rapidly during early life. Following the introduction of solid food at weaning (~3 weeks). By around 7–14 weeks, the gut microbiota in rats reflects adult configuration with higher diversity and stability (16–18). While rodent models provide a powerful tool to uncover mechanistic links between dietary fiber, microbial community dynamics, and host responses, the observed differences in gut physiology and microbiota composition must be recognized. Therefore, findings in rodents should be validated in well-designed human intervention studies before translational recommendations are made (12).

This study addresses three fundamental questions: (i) What constitutes the microbiota in Wistar rat healthy individuals, focusing on stable and permanent members of the community without the influence of external factors such as dietary interventions? (ii) How does a specific dietary fiber derived from cava lees alter the microbiota in healthy rats during 2-week intervention period? (iii) If compositional shifts in the microbiota were observed, do these changes related with functional alterations in the gut ecosystem?

By exploring these questions, we aim to expand understanding of the gut microbiota’s role in health and its modulation through dietary interventions with bioactive compounds. The relative abundance of genes for SCFA synthesis, bile acid metabolism, tryptophan pathways, and antioxidant function are widely accepted as important functional biomarkers of a healthy gut microbiota. Their relative gene abundances, as determined by shotgun metagenomics, will provide a holistic view of microbiome health. Because all rats are healthy, we hypothesize that changes might be subtle but tend towards enhanced functional capacity and microbiota stability. Beta-glucans from cava lees supplementation may generally supports beneficial functions even in already healthy individuals.

## Materials and methods

### Study design

All experimental procedures were conducted in accordance with Directive 2010/63/EU of the European Parliament regarding the protection of animals used for scientific purposes. The study was approved by the Bioethics Committee of the University of Barcelona (IRB00003099) and performed using 24 Wistar rats (12 males and 12 females). The trial was not registered in a preclinical database. The RjHan: Wi Wistar rats used in this study were supplied by Janvier Labs (France). These outbred rats are maintained to ensure less than 1% inbreeding per generation, resulting in a genetically heterogeneous stock.

The rats were 7–8 weeks old at study initiation, after 1 week of acclimatization period prior to treatment. Animals were maintained in a conventional temperature-controlled environment (22–25°C; 12-h light/dark cycle); HEPA filtered air-condition, room air renovation of 20–30 per hour, and with free access to food and water. Animals were provided with standard RM1-Dietex SDS feed (Argenteuil, France) and autoclaved water ad libitum. RM1-Dietex nutritional content were 46% starch, 4.1% sugars, 14.4% protein, 2.7% fat and 4.7% fiber.

The rats were randomly divided into two groups: control (*n* = 12, with 6 males and 6 females) and treatment (*n* = 12, with 6 males and 6 females). Three animals were housed per standard cage. These cages will be labeled with the study letters (C, Control; T, Treatment), numbers of animals (individually identified by a permanent mark at the base of its tails). Animals were randomly assigned to experimental groups using Microsoft Excel. Each animal was first assigned a unique identifier in a spreadsheet. A random number was generated for each animal using the RAND() function, and the resulting list was sorted in ascending order of random values. Animals were then allocated sequentially to experimental groups (e.g., Control, Treatment) in equal numbers. The treatment group (cava lees diet) received a daily dose of 2,000 mg lees/kg body weight via gavage (10 mL/kg/day) for 14 days. Cava lees were freshly prepared daily in 0.5% (w/v) aqueous carboxymethyl cellulose (CMC) solution (Panreac, Castellar del Vallès, Spain) before administration. The control group received only the CMC aqueous solution. Fecal samples were collected from both groups at days 0, 7, and 14. Upon arrival at the animal facility, all animals were weighed and underwent an initial veterinary health assessment. Throughout the study, animals were monitored daily by study personnel for food and water intake, clinical signs, and body weight. All animals completed the study without any adverse events.

Cava lees provided kindly by an alcoholic beavery Company were centrifuged at 10,000 rpm for 10 min at 4°C to remove residual cava. The lees were then frozen in an ultra-low temperature freezer (−80°C), freeze-dried (Cryodos-50, Telstar, Terrassa, Spain), ground, and stored in sealed tubes protected from light and humidity until use. The second fermentation lees of cava originate from an autolysis process in which most of the cytoplasmic material has been lost, implying that the samples are composed primarily of cell walls. Previously analysis of cell wall cava lees (Thesis, https://www.tdx.cat/handle/10803/399910#page=1; in Spanish), revealed a polysaccharide content of 76.2% and protein content of 9.2%.

### DNA extraction, shotgun sequencing and bioinformatic analyses

Bacterial DNA was extracted from fecal samples using a QIAamp PowerFecal DNA Kit (QIAGEN, MD, USA) according to the manufacturer’s protocol. DNA concentration was quantified using a BioDrop μLite spectrophotometer (Biotech, Madrid, Spain). Shotgun metagenomics analysis was performed using the Illumina MiSeq_RunMicro300 cycles (2 × 150) platform at the Genomic and Bioinformatic Service of the Autonomous University of Barcelona. *Paired-*end fastq files containing sequences generated by Illumina MiSeq_RunMicro300 cycles were assembled using the MEGAHIT tool (19) in Galaxy pipeline Version 1.2.9^1^ (20). The assembled sequences were submitted to the DOE-JGI Metagenome Annotation Pipeline (MAP v.4)^2^ (21), which employs the Velvet algorithm package for genomic assembly and short-read sequence alignment. Protein-coding genes were identified using a consensus of four different ab initio gene prediction tools: prokaryotic GeneMark.hmm (v.2.8), MetaGeneAnnotator (v. Aug 2008), Prodigal (v. 2.6.2), and FragGeneScan. Predicted protein-coding sequences shorter than 32 amino acids were excluded from further analysis. KEGG Orthology (KO) assignments were made when 90% of the gene sequence aligned with the reference database (21).

### Post-bioinformatics analyses

Alpha diversity was measured by Shannon index. Shannon’s index *H* is an estimator of taxa diversity, combining richness and evenness, were computed in R using the vegan package (22). Bray–Curtis dissimilarity analysis was applied to measure beta diversity and to generate principal coordinate analysis plots using the web-based tool JGI genomes online database (see text footnote 2). Differences in the microbiota composition between two groups were analyzed by Mann–Whitney-Wilcoxon test for non parametric data. Differences were considered significant with *p* < 0.05.

## Results

### Healthy Wistar rat baseline microbiota

**Figure 1:**
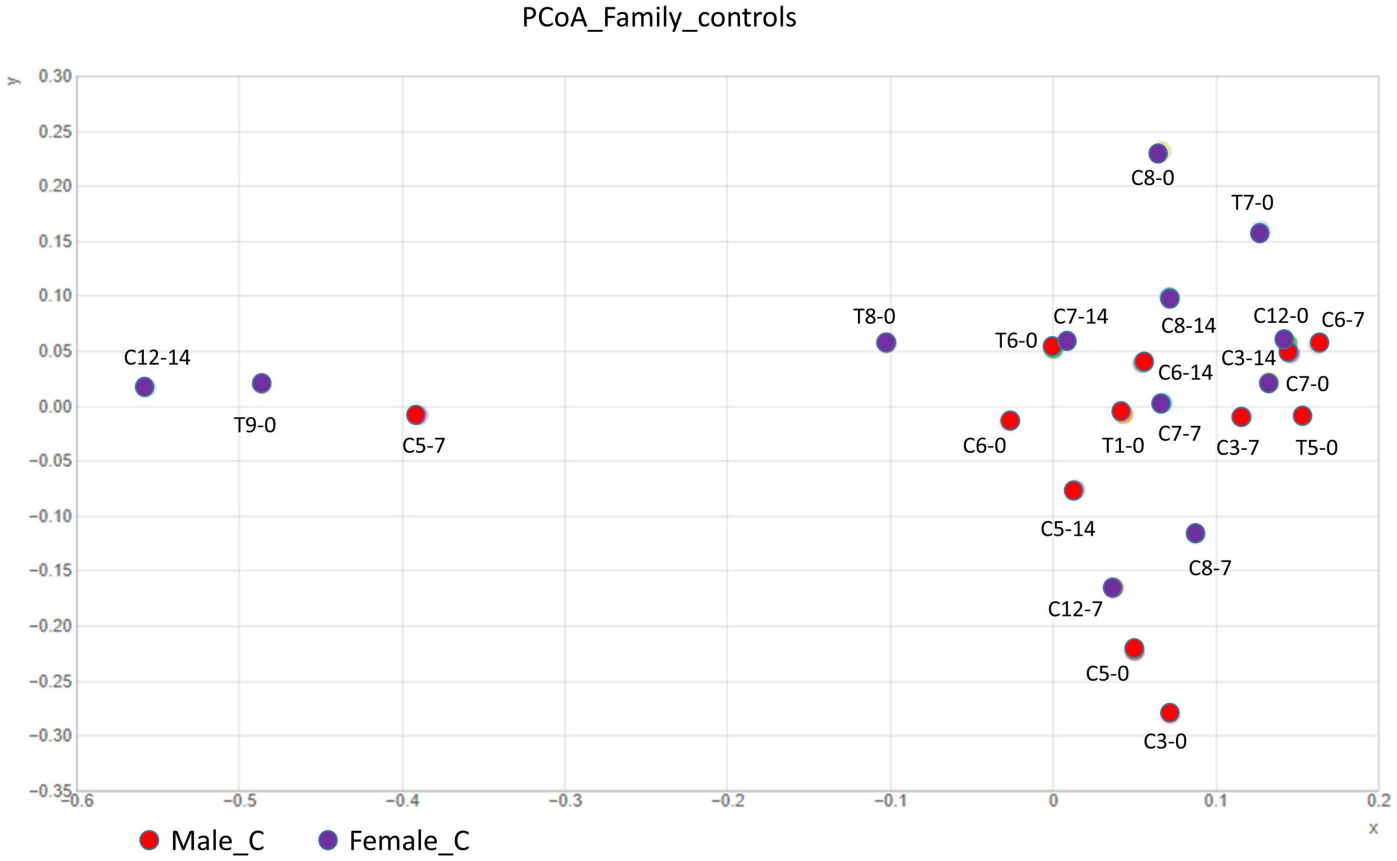
Principal coordinate analysis (PCoA) of microbial community distributions in healthy Wistar rats, comparing male (red circles) and female (purple circles) control groups. Sample labels follow this format: C indicates control group; the first number after C identifies individual rats; subsequent numbers (0, 7, or 14) indicate sampling days throughout the 14-day experiment. T, represents the treatment group, followed by rat identification number and 0 for initial sampling. Beta diversity was assessed using Bray–Curtis dissimilarity matrices (*p* > 0.05).

**Figure 2:**
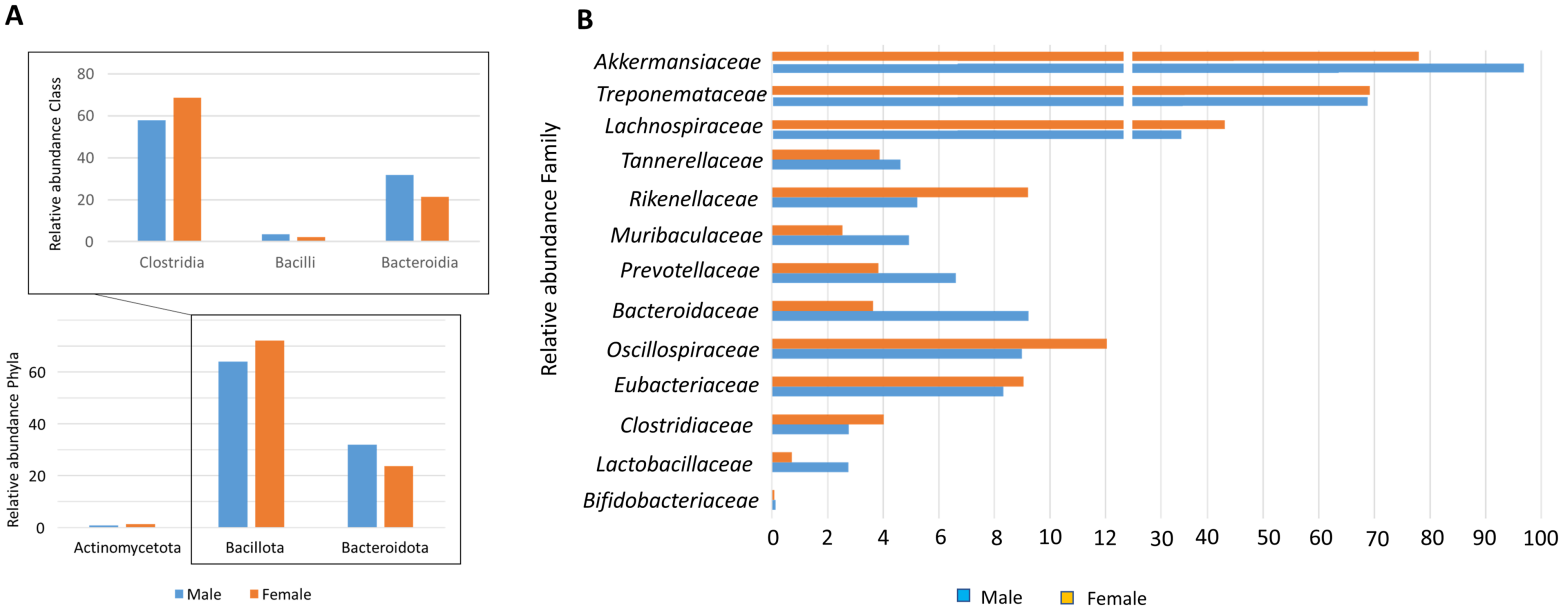
**(A)** Relative abundance of bacterial phyla and class in control group from male and female healthy Wistar rats. **(B)** Relative abundance of bacterial families in control group from male and female healthy Wistar rats.

The bacterial gut composition showed no significant differences between male and female Wistar rats at the global community level, although individual variations were observed (Figure 1). The healthy microbiota comprised more than 9 phyla, with Bacillota (64% in males and 72% in females) and Bacteroidota (32% in males and 23% in females) being the most abundant, while Actinomycetota represented only 1%. The remaining phyla showed minor relative abundance (<1%) in both male and female rats (Figure 2A) such as Fibrobacterota, Fusobacteriota, Pseudomonadota, Spirochaetota, Thermodesulfobacteriota, and Verrucomicrobiota. Despite the absence of significant differences in the overall community structure, analysis at the family level revealed sex-specific variations within both major and minor phyla. Male rats exhibited higher relative abundances of *Lactobacillaceae*, *Bacteroidaceae*, *Muribaculaceae*, and *Akkermansiaceae* compared to females. Conversely, female rats showed higher relative abundances of *Oscillospiraceae* and *Rickenellaceae* (Figure 2B).

### Healthy Wistar rat microbiota in short-term cava lees intervention

**Figure 3:**
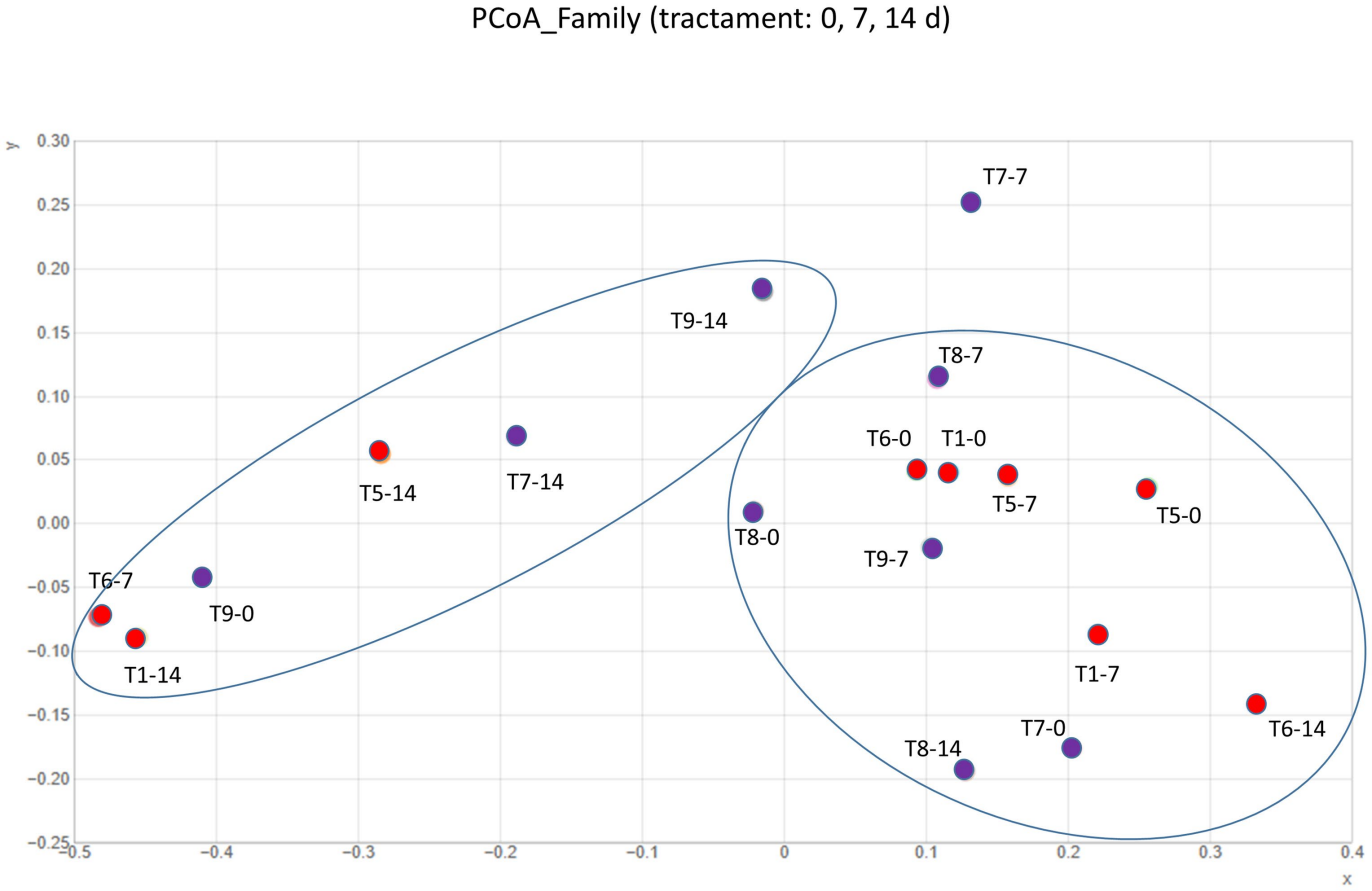
Principal coordinate analysis (PCoA) at family taxonomic level revealed distinct microbial community distributions in male (red circles) and female (purple circles) Wistar rats under treatment conditions. Sample labels follow the format: T indicates treatment group, followed by rat identification number and subsequent numbers (0, 7, or 14) indicate sampling days throughout the 14-day experiment. Microbial communities showed significant shifts from baseline (*T* = 0) to the end of treatment (*T* = 14) in all individuals analyzed. The analysis revealed two distinct clusters across both male and female rats: one comprising samples from days 0 and 7, and another consisting of samples collected after 14 days of treatment. Beta diversity was assessed using Bray–Curtis dissimilarity matrices (*p* < 0.05).

**Figure 4:**
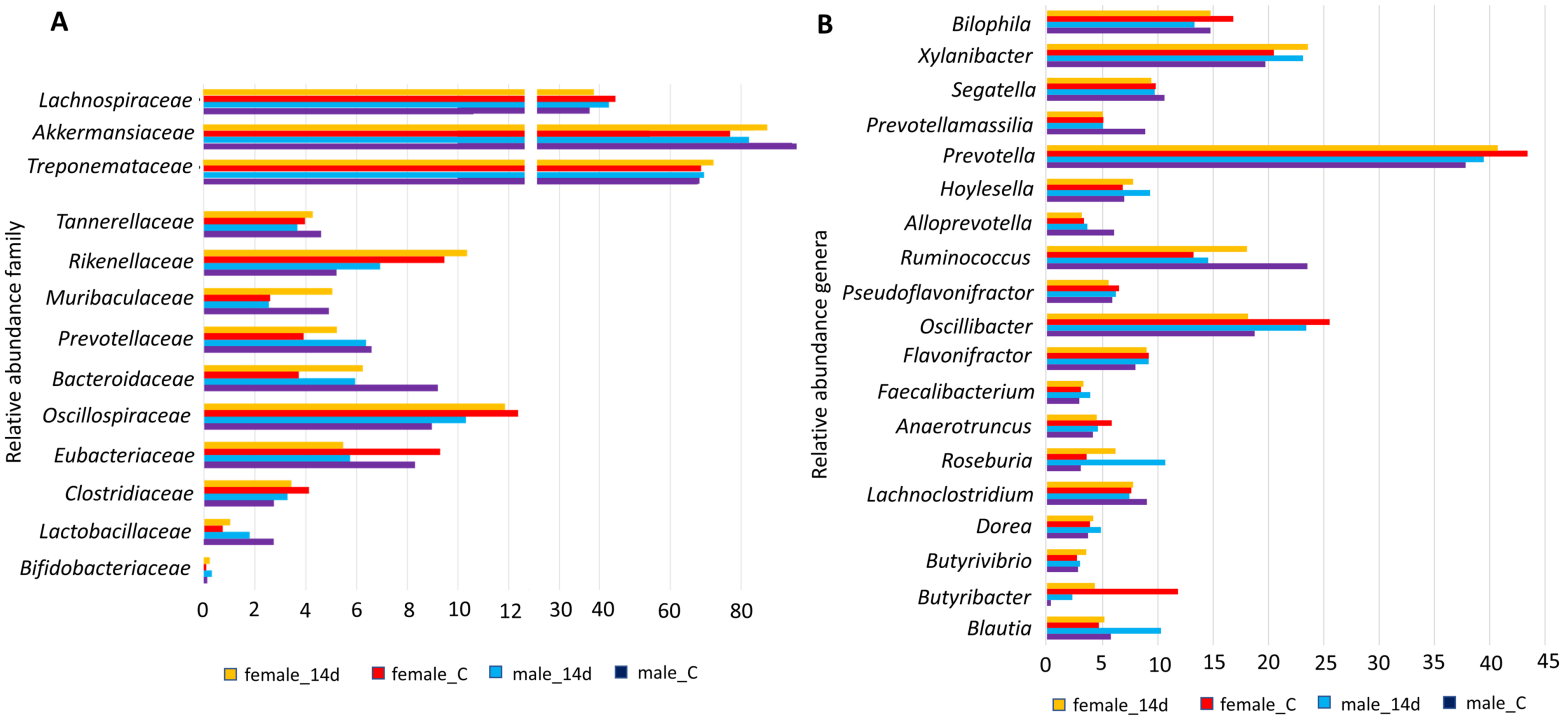
**(A)** Relative abundance of predominant bacterial families within major phyla in treated male and female Wistar rats. **(B)** Distribution of bacterial genera and their relative abundances across treatment groups in male and female Wistar rats.

Short-term intervention with cava lees showed changes in microbial composition for both male and female microbiota after a 14-day treatment compared to the control group or the 7-day treatment (Figure 3). The Shannon diversity index was increased in rats receiving the beta-glucans supplement. Shannon index in control were 5,804 and 5,171 for male and female respectively, but in the treatment, group were 6,192 and 6,552 for male and female, respectively (Mann–Whitney-Wilcoxon test comparing Shannon indexes between the control and treatment male rats groups *p* = 0.0089; and for female rats groups, *p* = 0.0043). Changes in microbiota seemed depend on the sex such as in the case of *Oscillspiraceae*, *Bacteriodaceae*, *Akkermansiaceae* or *Lachnospiraceae*. However, male and female increased in *Bifidobacteriaceae*, *Rikenellaceae* and decreased in *Eubacteriaceae*. Similar results was observed previously although there were not distinction between sex microbiota (23) (Figure 4A). At the genera level, distinct variations were evident in *Blautia*, *Butirybacter*, *Roseburia*, *Ruminococcus*, *Xylanibacter*, and *Bilophila*, highlighting the complex interactions between beta-glucans supplementation and gut microbiota. (Figure 4A). These results suggest that dietary supplements may influence gut microbial diversity and composition.

### Healthy Wistar rat microbiota functionality in control and short-term cava lees intervention

**Figure 5:**
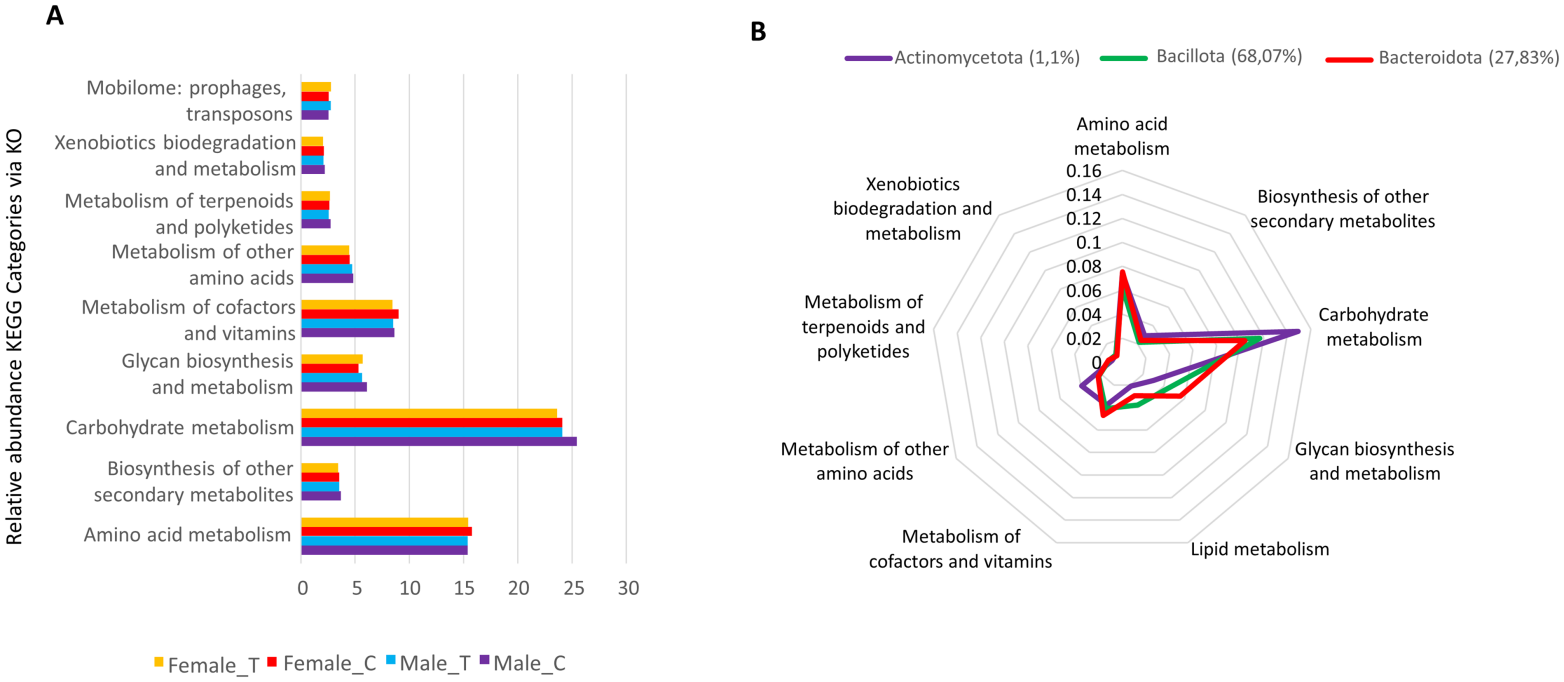
**(A)** Relative abundances of KEGG Orthologs (KOs) associated with major metabolic pathways, including carbohydrate metabolism, amino acid metabolism, and cofactor/vitamin metabolism. **(B)** Radial plot showing the proportional contributions of dominant bacterial phyla to key metabolic functions, including carbohydrate, amino acid, lipid, cofactor/vitamin, and xenobiotic metabolism.

Shotgun metagenomics was employed to elucidate the functional potential of the Wistar rat gut microbiota. Predicted proteins were systematically classified into Kyoto Encyclopedia of Genes and Genomes (KEGG) orthologs (KOs). The microbial functional profile primarily encompassed amino acid metabolism, carbohydrate metabolism, and metabolism of vitamins and cofactors. To a lesser extent, the microbiota also contributed to terpenoid/polyketide metabolism and xenobiotic degradation (Figure 5A). No statistically significant differences were observed in global metabolic profiles between the control and cava lees supplementation groups. Carbohydrate and amino acid metabolic processes were predominantly performed by Actinomycetota, Bacteroidota, and Bacillota, with lipid metabolism predominantly attributed to Bacillota (Figure 5B).

Although, in general, no differences were observed, if we look for specific functionalities we can detect changes in the relative abundance of some genes, which could be related to the observed changes in microbiota. To characterize carbohydrate-degrading enzymes, we analyzed Carbohydrate-Active Enzymes (CAZymes). The relative abundance of CAZymes involved in degrading cellulose, starch, trehalose, sucrose, pectin, arabin, galactan, and xylan were 7.32% in the control group versus 6.59% in the treatment group (Mann–Whitney-Wilcoxon test *p* = 0.197). Polysaccharides in cava lees cell walls contained β-1,3-glucans and β-1,6-glucans, and lower proportion of chitin. We specifically examined the relative abundance of beta-glucosidase gene-enzymes (EC:3.2.1.21, EC:3.2.1.58, and EC:3.2.1.86), which remained consistent at 1.43% in the control group and 1.36% in the beta-glucans supplementation group (Mann–Whitney-Wilcoxon test *p* = 0.401). The (nonsignificant) increase in chitinase enzyme abundance—from 0.48 to 0.57%—suggests metabolic responses to supplementation group (both in male and female rats) (Mann–Whitney-Wilcoxon test *p* = 0.941), potentially reflecting the activation or outgrowth of chitin-metabolizing microorganisms. Chitinase activity is generally associated with taxa like Actinomycetota and Bacteroidota (24).

The relative abundance of key genes-enzymes associated with butyrate production (EC:2.8.3.8; EC:2.8.3.9; EC:2.7.2.7; EC:2.3.1.16; EC:4.2.1.55; EC:1.1.1.157) were significant increased after treatment with cava lees (Mann–Whitney-Wilcoxon test *p* = 0.05). The abundance rose from 0.089% in the control group to 0.127% in the treatment group, with this increase observed consistently in both male and female rats. The approximately 40% increase in relative abundance suggests that the treatment may have selectively stimulated the metabolic activity or proliferation of butyrate-producing bacterial strains. Key propionate-related genes-enzymes (EC:5.4.99.2; EC:2.8.3.5; EC:2.7.1.17; EC:2.7.1.53) showed a modest (non-significant) increase of 15.7% in the treatment group compared to the control in both male and female rats (Mann–Whitney-Wilcoxon test *p* = 0.310). Genes related to acetate production (EC:5.4.99.2; EC:2.8.3.5; EC:2.7.1.17; EC:2.7.1.53) exhibited no significant change, remaining relatively stable between the control group (0.386%) and the treatment group (0.379%) (Mann–Whitney-Wilcoxon test *p* = 0.941).

Bile acids serve as critical mediators in the complex interplay between the gut microbiome and host metabolic processes. While primary bile acids originate in the liver, they undergo subsequent transformation into secondary bile acids through the metabolic activities of gut bacteria. Our analysis revealed an increase in the relative abundance of genes associated with secondary bile acids modification in the treatment group compared to the control group (0.108% vs. 0.141%). But only in the treatment female group was significant (Mann–Whitney-Wilcoxon test *p* = 0.032 versus male treatment group, Mann–Whitney-Wilcoxon test *p* = 0.069), suggesting a potential sex-specific response to the intervention.

Enzymes that catalyze the metabolism of amino acids and related compounds are responsible for generating several catabolites including p-cresol (transformed in the liver to p-cresyl sulfate), indole [transformed by the liver to (indoxyl sulfate), and trimethylamine (precursor of trimethylamine-N-oxide)]. Analysis of enzymatic profiles revealed no significant changes (Mann–Whitney-Wilcoxon test, *p* = 0.28) in the relative abundance of three key enzymes (EC:4.1.99.1, EC:4.1.99.2, and EC:3.1.6.6) in both male and female treatment groups compared to control rats.

Antioxidant activity in cava lees appears to be primarily associated with their protein fraction components (25). We investigated whether these antioxidant properties could modulate oxidative stress responses in intestinal microbiota. Our analysis revealed a reduction in the relative abundance of genes related to oxidative stress (including EC:1.11.1.21, EC:1.11.1.24, and EC:1.11.1.6) in subjects receiving the cava lees treatment compared to the control group. This reduction was statistically significant in males but not in females (Mann–Whitney-Wilcoxon test *p* = 0.016 and 0.1, respectively), suggesting potential sex-specific responses. Additionally, we observed decreased proportions of other oxidative stress-related genes in the treatment group, including those involved in: Glutathione metabolism (EC:2.3.2.2, EC:3.4.19.13, EC:6.3.2.2, EC:6.3.2.2/6.3.-.-, EC:1.8.1.7) and ferroptosis pathways (EC:2.3.1.57). However, these additional reductions did not reach statistical significance in either sex.

## Discussion

### Healthy Wistar rat microbiota control and treatment

Healthy Wistar rats, aged 7–8 weeks and presumed to have mature adult microbiota, with equal numbers of males and females, were subjected to a short treatment with cava lees. The choice of a 14-day intervention was based on prior studies in which short-term dietary interventions can induce measurable shifts in rodent gut microbiota composition and metabolic output (26–28). Nevertheless, we recognize that longer intervention periods could offer further insights into the persistence and adaptation of microbiota changes. Future studies should explore whether the altered gut microbiota profile is sustained after discontinuing beta-glucan supplementation, given that microbiota shifts are known to be reversible once dietary stimuli are withdrawn (29).

The baseline characterization of gut microbiota in healthy Wistar rats was dominated by the phyla Bacillota and Bacteroidota, consistent with established patterns in rodent gut colonization, where these bacterial groups play key roles in nutrient metabolism and immune system development. Although the global community structure showed no significant sex-based differences, a more detailed examination at the family level uncovered distinct sexual dimorphism. The observed sexual dimorphism at the family level, particularly the male-specific enrichment of *Lactobacillaceae*, *Bacteroidaceae*, *Muribaculaceae*, and *Akkermansiaceae*, suggests potential hormone-mediated influences on microbial colonization patterns (30). These bacterial families are known to contribute to various physiological processes, including carbohydrate metabolism, barrier function maintenance, and immune system modulation (31). The female-specific elevation of *Oscillospiraceae* and *Rickenellaceae* further underscores the complex interplay between host physiology and microbial community structure.

The introduction of cava lees as a dietary intervention highlights the remarkable plasticity of the gut microbiome and its responsiveness to dietary modifications (32, 33). The enhanced microbial diversity following cava lees beta-glucan supplementation, particularly pronounced after 14 days, indicates the prebiotic potential of these compounds. Beta-glucans, known for their immunomodulatory properties, appear to create a favorable environment for beneficial bacteria, as evidenced by the proliferation of *Bifidobacteriaceae*, which are associated with improved gut barrier function and enhanced immune response. The sex-specific modulation of certain bacterial families, including *Oscillspiraceae*, *Bacteriodaceae*, *Akkermansiaceae*, and *Lachnospiraceae*, points to the existence of sex-dependent mechanisms in microbiota-diet interactions. Male and female rats have different hormonal profiles (e.g., estrogen, testosterone), which may influence the immune system, metabolism, and gut physiology. These, in turn, affect gut microbiota composition and function. Several studies in rats showed sex-dependent differences in gut microbiome composition, microbial richness, and function as our results (34–36).

*Lachnospiraceae*, *Oscillospiraceae* and *Bifidobacteriaceae* were involved in the production of short-chain fatty acids (SCFAs), particularly butyrate, which serves as an energy source for colonocytes and exhibits anti-inflammatory properties (37). *Akkermansiaceae* were associated with overall SCFA metabolism and mucin degradation. Generally, *Bacteroidaceae* are more closely linked to the production of acetate and propionate rather than butyrate (38). At the genus level, the modifications in *Blautia*, *Butirybacter*, *Roseburia*, *Ruminococcus*, *Xylanibacter*, and *Bilophila* reflect the broad impact of beta-glucans on diverse bacterial populations involved in fiber fermentation, SCFA production, and various metabolic pathways. A statistically significant reduction in the relative abundance of *Bilophila* was observed following the intervention, with Mann–Whitney-Wilcoxon test *p*-values of 0.016 for male rats and 0.0079 for female rats. This finding is particularly important because elevated levels of *Bilophila* have been previously linked to inflammation and metabolic disorders in both healthy and high-fat diet mouse models (23). *Bilophila wadsworthia*, in particular, is known to promote inflammatory processes through the production of hydrogen sulfide, which can damage the intestinal barrier and trigger immune responses. Therefore, the observed decrease in *Bilophila* abundance suggests a potentially beneficial impact of the intervention on gut health, as it may help reduce pro-inflammatory signaling and lower the risk of metabolic disturbances associated with this bacterial genus.

### Healthy Wistar rat microbiota functionality

Shotgun metagenomic analysis of the Wistar rat gut microbiome unveiled a complex functional landscape dominated by essential metabolic pathways. The primary functional capabilities encompassed amino acid metabolism, carbohydrate metabolism, and vitamin/cofactor synthesis, reflecting the fundamental role of gut microbiota in host nutrition and health (9, 39) The taxonomic distribution of these metabolic functions demonstrated specialization among major phyla, with Actinomycetota, Bacteroidota, and Bacillota handling most carbohydrate and amino acid processes.

The examination of Carbohydrate-Active Enzymes (CAZymes) provided insights into the microbial community’s capacity to process various dietary components (40). Beta-glucan yeast supplementation did not significantly alter the abundance of beta-glucosidases, suggesting the microbiota maintains stable carbohydrate-processing capacity even when dietary carbohydrates are introduced. These stable abundance and activity of beta-glucosidases during dietary beta-glucan supplementation aligned with the concept of functional redundancy in the gut microbiome. Multiple microbial species may contribute to the community’s enzymatic reservoir, ensuring the complex carbohydrate degradation (41–44).

The enhanced genetic potential for butyrate production following beta-glucan supplementation suggests a beneficial shift in the gut metabolic profile, as butyrate plays crucial roles in maintaining intestinal barrier integrity, reducing inflammation, and supporting colonocyte health. *Roseburia*, *Ruminococcus, Blautia, Butirybacter* and *Bifidobacterium* are well-established butyrate-producing genera (45). The modest increase (non-significant) in propionate-related genetic capacity, coupled with stable acetate production potential, suggested a selective modulation of SCFA metabolism. This metabolic shift could have implications for host health, given the diverse physiological roles of SCFAs, including energy metabolism, immune regulation, and gut-brain communication.

Also, our findings revealed an enhanced genetic capacity for secondary bile acid modification, particularly pronounced in female subjects, indicating sex-specific responses in microbial metabolism (Mann–Whitney-Wilcoxon test *p* = 0.032). This sexual dimorphism in bile acid processing has potentially far-reaching implications for host metabolism, considering the crucial role of bile acids as signaling molecules in glucose homeostasis, lipid metabolism, and energy expenditure (46). These sex differences in bile acid metabolism may contribute to the differential effects of dietary interventions like yeast beta-glucan. Baars et al. (47) demonstrated that the presence of gut microbiota is specifically required for sex-specific differences in lipid metabolism to be observed. While germ-free male and female mice still exhibit biological differences, they do not show distinctions in fat processing mechanisms. Only when gut bacteria are present do the sex-specific patterns in lipid metabolism become apparent. In conventional mice with normal gut bacteria, the most significant differences between sexes were observed in genes related to lipid metabolism, whereas germ-free mice showed differences primarily in genes involved in gut health and inflammatory responses.

Microbial tryptophan catabolites derived from proteolytic activity play a complex role in host health, acting as both potential uremic toxins and beneficial compounds within the intestinal environment (48). Typically, increased production of metabolites such as p-cresol and indole is linked to specific bacterial genera that possess enzymes involved in amino acid metabolism, including *Clostridium*, *Bacteroides*, and *Desulfovibrio* (49). In our study, although the relative abundance of Clostridium and Bacteroides increased in both male and female treatment groups, enzymatic profiling showed no significant changes in the relative abundance of key enzymes (EC:4.1.99.1, EC:4.1.99.2, and EC:3.1.6.6) responsible for catabolizing amino acids into compounds like p-cresol, indole, and trimethylamine (Mann–Whitney-Wilcoxon test, *p* = 0.28). Therefore, our results highlight that short-term dietary modulation with cava lees can shift the relative abundance of key microbial taxa without significantly altering enzymatic activity linked to potentially harmful amino acid catabolites.

The antioxidant activity found in cava lees was linked to their protein fraction, which could modulate oxidative stress responses within the intestinal microbiota. Intestinal bacteria are continually exposed to oxidative challenges that stem from three primary sources: host-derived oxidants (such as reactive oxygen species and reactive nitrogen species produced by the intestinal epithelium for immune control), dietary oxidants, and the presence of inflammation, all of which elevate levels of damaging reactive species in the gut environment (50). Protein-based antioxidant compounds—including those from foods or cava lees—can trap free radicals, neutralize reactive oxygen species (ROS) and reactive nitrogen species (RNS), and ultimately support the survival and balance of beneficial gut bacteria, which is essential for gut health and maintaining epithelial barrier function (51). Our results showed that treatment with cava lees significantly reduced oxidative stress in the intestinal bacteria of male rats. However, this antioxidant effect was not observed in the female group. Gender differences (as in human studies) may influence oxidative stress responses, with males generally being more susceptible to oxidative stress and less efficient in antioxidant defense compared to females (52).

## Conclusions and perspectives

The effects of yeast beta-glucan supplementation on the Wistar rat gut microbiome had open insights and future research directions. The study showed that baseline gut microbiota exhibits sexual dimorphism at the family level. Dietary beta-glucans intake induces selective changes in the microbial community structure and function. Notably, the increased genetic potential for butyrate production and the sex-specific modulation of bile acids metabolism and oxidative stress highlights the complex interplay between dietary interventions and host physiology.

Future research directions should focus on several key areas to expand these findings. Longitudinal studies using varying beta-glucan concentrations and longed intervention periods could provide deeper insights into how the microbiota adapts over time. Employing multi-omic approaches, such as metatranscriptomics and metabolomics, would offer a more comprehensive understanding of the functional implications of observed taxonomic shifts. Additionally, examining the potential therapeutic applications of targeted beta-glucan supplementation in different metabolic disorders.

This work underscores the importance of considering sex-specific responses in microbiome research and highlights the potential of dietary interventions in modulating gut microbial functionality. The findings provide a foundation for future studies aimed at exploring the therapeutic potential of beta-glucans and similar dietary compounds for promoting gut health and managing metabolic disorders through microbiome modulation.

## Data Availability

The original contributions presented in the study are publicly available. This data can be found at: https://gold.jgi.doe.gov/study?id=Gs0142379.

## Footnotes

^1^ https://usegalaxy.eu/

^2^ https://gold.jgi.doe.gov/
